# Phytochemical Profiling of Flavonoids, Phenolic Acids, Terpenoids, and Volatile Fraction of a Rosemary (*Rosmarinus officinalis* L.) Extract

**DOI:** 10.3390/molecules21111576

**Published:** 2016-11-19

**Authors:** Pedro Mena, Martina Cirlini, Michele Tassotti, Kelli A. Herrlinger, Chiara Dall’Asta, Daniele Del Rio

**Affiliations:** 1 Department of Food Science, University of Parma, Parma 43125, Italy; pedromiguel.menaparreno@unipr.it (P.M.); martina.cirlini@unipr.it (M.C.); michele.tassotti@studenti.unipr.it (M.T.); chiara.dallasta@unipr.it (C.D.A.); 2 Kemin Foods, L.C., 2100 Maury Street, Des Moines, IA 50317, USA; kelli.herrlinger@kemin.com

**Keywords:** rosemary, polyphenol, volatile compound, phytochemical characterization, UHPLC-ESI-MS^n^, HS-SPME/GC-MS

## Abstract

This paper presents a comprehensive analysis of the phytochemical profile of a proprietary rosemary (*Rosmarinus officinalis* L.) extract rich in carnosic acid. A characterization of the (poly)phenolic and volatile fractions of the extract was carried out using mass spectrometric techniques. The (poly)phenolic composition was assessed by ultra-high performance liquid chromatography-electrospray ionization-mass spectrometry (UHPLC-ESI-MS^n^) and a total of 57 compounds were tentatively identified and quantified, 14 of these being detected in rosemary extract for the first time. The rosemary extract contained 24 flavonoids (mainly flavones, although flavonols and flavanones were also detected), 5 phenolic acids, 24 diterpenoids (carnosic acid, carnosol, and rosmanol derivatives), 1 triterpenoid (betulinic acid), and 3 lignans (medioresinol derivatives). Carnosic acid was the predominant phenolic compound. The volatile profile of the rosemary extract was evaluated by head space solid-phase microextraction (HS-SPME) linked to gas chromatography-mass spectrometry (GC-MS). Sixty-three volatile molecules (mainly terpenes, alcohols, esters, aldehydes, and ketones) were identified. This characterization extends the current knowledge on the phytochemistry of *Rosmarinus officinalis* and is, to our knowledge, the broadest profiling of its secondary metabolites to date. It can assist in the authentication of rosemary extracts or rosemary-containing products or in testing its bioactivity. Moreover, this methodological approach could be applied to the study of other plant-based food ingredients.

## 1. Introduction

Rosemary (*Rosmarinus officinalis* L.), which belongs to the family Lamiaceae, is an aromatic, evergreen, 1-m high shrub with upright stems, whitish-blue flowers and dark green leaves. The foliage of the plant is usually used as a common household culinary spice for flavoring [1,2,3]. Rosemary extracts, mainly derived from the leaves, are common herbal products used as flavoring and antioxidant agents in food processing and cosmetics. As naturally occurring antioxidants, they are preferred to synthetic antioxidants such as butylated hydroxyanisole (BHA) or butylated hydroxytoluene (BHT) [4]. Moreover, rosemary has been used in traditional and complementary alternative medicine for its digestive, tonic, astringent, diuretic, and diaphoretic properties [1,2,3]. It has also been linked to a broad range of beneficial health effects, having for example antidepressant [5], antihypertensive [6], antiproliferative [7], antibacterial [8], antiatherogenic [9], hypocholesterolemic [10], hepatoprotective [11], and anti-obesity properties [11,12].

The biological properties of rosemary have been attributed to its phytochemical composition rich in (poly)phenolic compounds, mainly diterpenoids such as carnosic acid and carnosol [5,7,10,11,12]. However, the positive contribution of flavonoids to rosemary bioactivity is also reported in the literature [5]. After considering the co-presence of flavonoids and diterpenes in the plant [3,13], the way these compounds are metabolized [14,15,16], and their consequent co-occurrence in circulation, the benefits ascribed to rosemary cannot be unambiguously attributed to a single class of compounds, but rather to the multiple contribution of its different bioactive compounds. Furthermore, the phenolic composition of rosemary extracts has been reported to vary depending on agronomical and processing conditions [17,18,19,20]. For this reason, the phenolic fraction of every rosemary product should be accurately characterized to better understand its technological and bioactivity prospects. In addition, due to the contribution of the volatile profile of any food extract to its potential uses, the characterization of the volatile fraction of rosemary extracts should also be evaluated.

This study aimed to comprehensively profile the phytochemical composition of an extract rich in carnosic acid from a proprietary rosemary line. The (poly)phenolic composition was assessed by means of ultra-high performance liquid chromatography-electrospray ionization-mass spectrometry (UHPLC-ESI-MS^n^), whereas the volatile profile was studied using head space solid-phase microextraction/gas chromatography-mass spectrometry (HS-SPME/GC-MS).

## 2. Results and Discussion

### 2.1. Profiling of the Phenolic Composition

The (poly)phenolic profile of the proprietary rosemary extract rich in carnosic acid was evaluated using an UHPLC-ESI-MS^n^ untargeted method consisting of two complementary mass spectrometry (MS) conditions [21]. About 190 mass spectra were assessed for each analytical replicate and MS operating condition in this comprehensive approach for a complete screening of (poly)phenolic compounds. This procedure allowed a detailed evaluation of the rosemary extract phenolic fraction and the tentative identification of up to 57 phytochemicals (Table 1). The most represented classes of (poly)phenolic compounds in the extract were diterpenoids and flavonoids (flavones, flavanones, and flavonols), with a total of 24 molecules identified for each class. Some phenolic acids and lignans, as well as one triterpenoid, were also identified.

Table 1 describes the retention time and mass spectrum data for each identified compound. Ten compounds were identified and quantified by comparison with commercial reference standards. The identification of the remaining 47 (poly)phenolic compounds was tentatively carried out by interpreting and comparing their mass spectra, obtained from MS^2^ and MS^3^ experiments with data from the literature. Fourteen phytochemicals (compounds **13**, **14**, **15**, **16**, **18**, **20**, **27**, **29**, **36**, **38**, **40**, **43**, **50**, and **53**) were tentatively identified for the first time, to our knowledge, in rosemary extracts. The description of the MS fragmentation patterns already described in literature is not further discussed unless of special interest.

**Figure 1:**
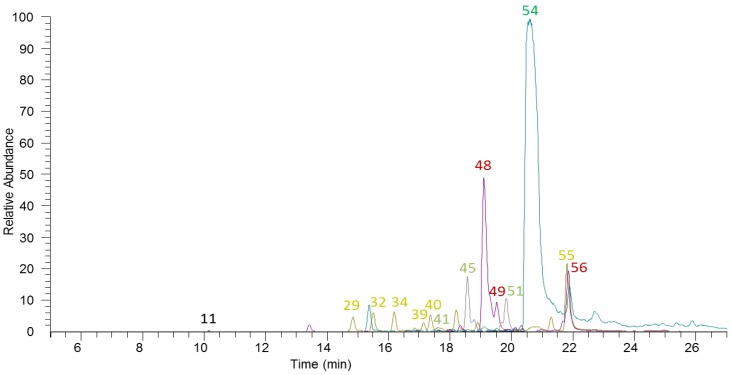
Main rosemary (poly)phenolic compounds. Peak numbers refer to components listed in Table 1.

A total of 24 diterpenoids were identified in the rosemary extract (Figure 1). Most of the detected diterpenoids had already been reported in rosemary (compounds **31**, **35**, **39**, **41**, **42**, **44**–**49**, **51**, **52**, and **54**–**56**) [3,4,12,13,17,18,23,29,32]. Compounds **31**, **33**, **41**, **42**, **45**, and **51** exhibited molecular ions at *m*/*z* 343. Carnosol methyl ether isomers (compounds **31**, **41**, and **42**) were distinguished from rosmadial or rosmanol quinone isomers (**33**, **45**, and **51**) thanks to the fragment ion at *m*/*z* 328 and the neutral loss of 15 amu, characteristic of the methyl group [14]. Unfortunately, distinction between rosmadial or rosmanol quinone isomers was not possible as they share a common fragmentation pattern. The presence in rosemary extracts of several isomers for these molecules has been previously reported [14]. Glycosylated carnosic acid (compound **38**) was tentatively identified through its MS^2^ fragment ions, characterized by the loss of a hexoside (162 amu), and MS^3^ fragments identical to those registered for the standard of carnosic acid (**54**). This approach was also used to identify another carnosic acid derivative (compound **43**). Compounds **29** and **40** (*m*/*z* 345) were tentatively identified as derivatives of 5,6,7,10-tetrahydro-7-hydroxyrosmariquinone. They fragmented to *m*/*z* 301 (with neutral loss of 44 amu, likely corresponding to a carboxylic group from the parent ion) and their MS^3^ fragmentation spectra matched the characteristic fragmentation pattern of 5,6,7,10-tetrahydro-7-hydroxyrosmariquinone (compound **53**) [12]. These two derivatives had only been previously described in biological fluids of rats following the intake of a rosemary extract [14].

Twenty-four flavonoids, belonging to three subclasses of flavonoids (flavones, flavonols, and flavanones), were tentatively identified. Flavones were the main group of flavonoids in the rosemary extract, with 17 compounds identified. Nine of these were conjugated forms (mainly glycosylated) of luteolin (compounds **4**, **5**, **12**, and **17**), apigenin (**8** and **25**), hispidulin (**10** and **27**), and a dihydroxy-dimethoxyflavone (**13**) [3,4,13,24]. A large number of flavone aglycones with different hydroxylation and/or methylation patterns was also detected (**14**, **16**, **21**, **23**, **26**, **30**, **37**, and **50**). The retention time and fragmentation pattern of compound **23** (*m*/*z* 299) did not match well with those already reported for other trihydroxy-methoxyflavones previously identified in rosemary extracts such as diosmetin or hispidulin [23]. With respect to flavanones, three aglycones (compounds **19**, **28**, and **36**) and one rutinoside (**9**) were detected. Isorhamnetin was the only flavonol detected, in both the free (**22**) and glycosylated forms (**6** and **20**) [4].

Five phenolic acids were identified in the rosemary extract, a hydroxybenzoic acid (compound **7**), two hydroxycinnamic acids (**1** and **3**), and two rosmarinic acid derivatives (**11** and **24**). These findings are in agreement with previous works [4,13]. The profiling of the (poly)phenolic fraction of the rosemary extract also allowed the identification of three lignans, namely medioresinol (**2**) [12] and two medioresinol derivatives (**15** and **18**), the latter tentatively identified for the first time in this plant material.

Only one triterpenic acid, betulinic acid (**57**), was detected. Oleanolic acid and ursolic acid, typically present in the triterpenoid fraction of rosemary [3], were not detected in this extract.

**Figure 2:**
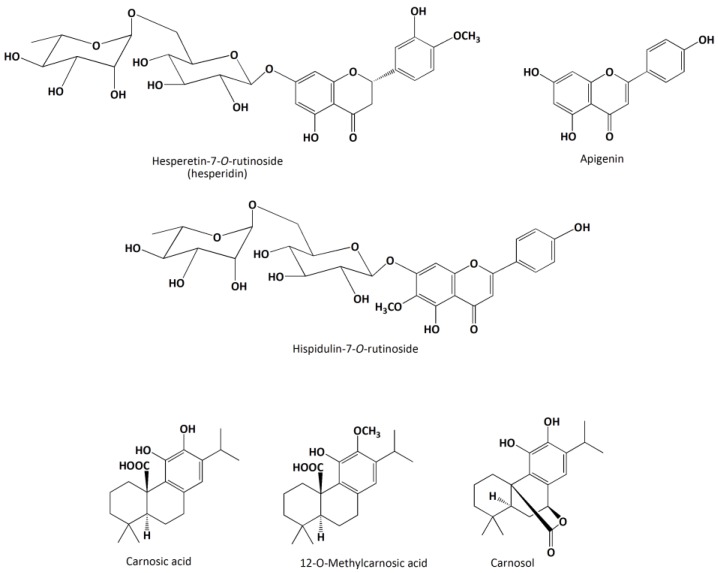
Some of the (poly)phenolic compounds present in the rosemary extract.

This comprehensive analysis of the phenolic composition of a rosemary extract represents the broadest characterization of its (poly)phenolic fingerprint to date. From the 57 (poly)phenolic compounds tentatively identified, a quarter corresponded to molecules not previously reported as present in this plant. Despite accurate characterizations of rosemary extracts reported in the literature [4,12,17,18,23,32], this work extends the range of molecules contributing to the definition of this food matrix, and may assist in the study of its bioactive properties (Figure 2).

The specific experimental condition in which each compound was detected is reported in Table 1. Interestingly, while some chemical scaffolds could not be identified under experimental condition 2 (optimized for rosmarinic acid analysis), all the structures responded well to the MS settings of experimental condition 1 (optimized for carnosol analysis). In comparison with some other works using the same methodology [21,33], this is the first time that a specific MS configuration was able to detect all the identified compounds of a phenolic-rich plant matrix. This information may account for the versatility of MS experimental condition 1 in identifying varying phenolic structures, such as simple phenolic acids, different kinds of flavonoids, diterpenoids, and triterpenoids.

The quantification of phenolics was carried out by comparison with commercial standards, when available. For those compounds that could not be quantified with their corresponding standards, a reference compound was selected based on structural similarity and considering the functional groups that may affect the ionisation properties (i.e., carnosol derivatives were quantified as carnosol, rosmanol derivatives as rosmanol, flavonols as rutin (quercetin-rutinoside), flavones as luteolin-4-glucoside, etc.). Finally, the molecules responding to the electro-spray ionisation (ESI) source in a unique way with respect to the reference compound of choice, or not reaching the limit of quantification of the corresponding reference compound, were not quantified.

The amount of (poly)phenolic compounds in this rosemary extract was 166.32 ± 11.05 mg/mL. Although the (poly)phenolic profile of the extract was composed of a high number of different phenolic structures (Table 1), diterpenoids accounted for the 97.2% of this phenolic content (161.66 ± 10.64 mg/mL). Furthermore, this was attributed mainly to the amount of carnosic acid derivatives in the extract (77.1% of total phenolics, Table 2). Flavonoids represented about 1.4% (2.38 ± 0.22 mg/mL) of the total amount of detected (poly)phenolic compounds, followed by triterpenoids (1.3% of total phenolics, 2.10 ± 0.25 mg/mL). Phenolic acids made up only 0.1% of the total phenolic fraction. The amount of phenolic compounds previously reported for other rosemary extracts was quite variable and ranged from ~39.3 mg/g [18] to 523 mg/g [12], with some extracts showing a similar content to that reported here [3]. In accordance with our data, other rosemary extracts were composed mainly of carnosic acid, followed by carnosol and other diterpenoids, with flavonoids as minor components [3,12,18]. It should be noted that the amount and relative contribution of each class of (poly)phenolic compounds to rosemary extracts have been reported to be dependent on the extraction procedure and solvent used [17,18,19]. In addition, irrigation conditions, harvest time, storage conditions, and drying treatments are also factors that may affect the final phenolic composition of rosemary extracts [18,20].

### 2.2. Volatile Profile of Rosemary Extract

The composition of the volatile fraction of rosemary extract was investigated by means of HS-SPME/GC-MS technique. The obtained profile was composed of 63 different gas-chromatographic signals. Two approaches were combined for peak identification: the comparison of registered mass spectra with those present in the instrument library (NIST 14), and the calculation of LRIs (linear retention index) obtained on two different stationary phase columns (SUPELCOWAX 10 and BP5MS). The relative amounts of all identified compounds were calculated based on comparison to an internal standard (toluene). Results are listed in Table 3.

The aromatic profile of the rosemary extract was composed of about 628 µg/g of volatile compounds. These results differed from those obtained by Szumny et al. [50], who reported a total volatile amount of 135 g/kg (135,000 µg/g) in a rosemary mixture of fresh leaves, branches, and stems. However, they also showed a decrease of 44% in volatiles during the rosemary drying process [50]. Therefore, it is possible that the lower volatile amount found in our sample may be attributed to both the drying and extraction procedure used. Moreover, it should be mentioned that the characteristic rosemary volatiles pertain to the terpene class, which are usually contained in the non-polar fraction of rosemary: the essential oil. It was demonstrated that different extraction methods, such as extraction with solvents (hexane-acetone), distillation, use of supercritical CO_2_ or microwaves, utilized on rosemary leaves to obtain the essential oil, lead to different yields in term of volatile percentage [51]. The solvent used for extraction of the rosemary sample in this study was focused on recovery of the (poly)phenolic fraction and not on the essential oil. For this reason, it seems reasonable to find a lower concentration of volatile compounds in contrast to other processes targeting the extraction of rosemary essential oil, or its volatile fraction.

As expected, the class of molecules that mainly contribute to the volatile profile of rosemary extract are the terpenes (primarily mono- and sesquiterpenes), with more than 40 peaks representing 90% of the total volatile amount, followed by alcohols and esters (4% of total volatiles), and aldehydes (3% of total volatiles), as shown in Table 3. Small amounts of some ketones, one furan, and other non-fully identified compounds were also detected.

Among terpenes, verbenone and α-thujene were the most abundant compounds, in combination representing 24% of the total volatiles (77.59 ± 12.85 µg/g and 76.26 ± 13.13 µg/g, respectively). They were followed by bornyl acetate (54.02 ± 8.77 µg/g), camphor (41.52 ± 6.00 µg/g), α-caryophyllene (38.53 ± 7.24 µg/g), p-cymenene (34.70 ± 5.71 µg/g), β-caryophyllene (26.44 ± 4.84 µg/g), α-terpineol (24.70 ± 4.46 µg/g), and eucalyptol (20.22 ± 2.58 µg/g). All of these molecules contributed to give woody, camphoreous, mentholic, and phenolic aromatic notes to the rosemary extract. Our results were in agreement with those already reported in literature for rosemary essential oil, in which terpenes represented the prevalent compounds of the volatile profile. Li et al. [52] investigated the volatile composition of rosemary essential oils extracted from 18 different rosemary cultivars collected from the Mediterranean area, and found a prevalence of terpenes in the volatile fractions of all the selected rosemary cultivars. In particular, α- and β-pinene and myrcene emerged among the monoterpene hydrocarbons, while 1,8-cineol (eucalyptol), camphor, verbenone, and bornyl acetate were the prevalent compounds in the oxygenated monoterpenes sub-group [52]. Many compounds detected in the volatile fraction of this rosemary extract were also identified in Brazilian rosemary essential oil by Lemos et al. [53], who further demonstrated that the volatile fraction of rosemary could depend on seasonality. In 2012, Lakušić et al. [54] demonstrated the existence of two major oil chemotypes while studying the chemical composition of rosemary essential oil from the Balkan peninsula. One chemotype was characterized by the predominance of camphor in the aromatic fraction, while a second was defined by the predominance of 1,8-cineol (eucalyptol) [54]. Similarly, rosemary chemotypes characterized by verbenone, 1,8-cineol, and camphor, or by verbenone and α-pinene as major constituents have been identified and associated with geographical origin and climatic conditions of growth [55,56]. In the current study, a prevalence of verbenone (77.59 ± 12.85 µg/g), camphor (41.52 ± 6.00 µg/g), and lower concentrations of eucalyptol (20.22 ± 2.58 µg/g) were recorded. Thus, it is possible that the rosemary line utilized may be related to a chemotype in which verbenone and camphor are preferably bio-synthesized. Besides verbenone and camphor, considerable amounts of borneol and α-pinene were observed (11.92 ± 2.01 µg/g and 4.34 ± 0.65 µg/g, respectively). This is expected since they are major components of the rosemary aromatic profile [57,58]. On the contrary, camphene, a volatile compound typically present in rosemary, could not be identified among all the detected molecules.

Among minor compounds, small amounts of alcohols, esters, 2-phenyl ethanol and 2-phenylethyl acetate, were detected (1.00 ± 0.22 µg/g and 0.98 ± 0.12 µg/g, respectively). These compounds could confer floral aromatic notes to the sample, with the former associated with notes of rose. In addition, considerable amounts of ethyl caprylate and ethyl caprate were found (7.66 ± 2.34 µg/g and 12.41 ± 1.93 µg/g, respectively). These compounds are observed in other matrices, such as wine [39]. Finally, volatiles belonging to the aldehyde class, such as hexanal, heptanal, 2-heptenal, and nonanal, were also identified. Aldehydes have been recently reported as components in the volatile profile of rosemary essential oil extracted from *Rosmarinus eriocalyx* [59].

## 3. Materials and Methods

### 3.1. Materials

Acetonitrile, methanol, formic acid, caffeic acid, hesperidin, rutin, vitexin, C_8_-C_20_ alkane solution, and toluene were purchased from Sigma-Aldrich (St. Louis, MO, USA). Carnosic acid, carnosol, 12-*O*-methylcarnosic acid, rosmanol, rosmarinic acid, and betulinic acid were purchased from PhytoLab (Vestenbergsgreuth, Germany). Luteolin-4-glucoside was obtained from AASC Ltd. (Southampton, UK). Ultrapure water from MilliQ system (Millipore, Bedford, MA, USA) was used throughout the experiment. The proprietary rosemary extract rich in carnosic acid was provided by Kemin Foods, L.C. (Des Moines, IA, USA). It was prepared from dried leaves by a proprietary acetone-based extraction.

### 3.2. Identification and Quantification of (Poly)phenolic Compounds by UHPLC-ESI-MS^n^

The (poly)phenolic compounds in the sample were extracted according to previous reports [33,60], with some modifications. A mixture of 150 µL of extract and 1 mL of acetonitrile acidified with formic acid (2%) was ultrasonicated for 10 min and subsequently centrifuged at 10,480× *g* for 5 min at room temperature. The supernatant was directly injected into the UHPLC-MS system. Aliquots diluted with acidified acetonitrile (1/100 and 1/10,000) were also analyzed to quantify within the linearity range of the reference compounds, avoiding MS signal saturation. The sample was extracted in triplicate.

The extract of rosemary was analyzed using an Accela UHPLC 1250 equipped with a linear ion trap-mass spectrometer (LTQ XL, Thermo Fisher Scientific Inc., San Jose, CA, USA) fitted with a heated-ESI probe (H-ESI-II; Thermo Fisher Scientific Inc.). Separations were performed using a XSELECTED HSS T3 (50 mm × 2.1 mm), 2.5 µm particle size (Waters, Milford, MA, USA). The volume injected was 5 µL and the column oven was set to 30 °C. Two MS experiments were performed in negative mode [21].

An MS experiment optimized in negative mode for carnosol analysis (experimental condition 1) was carried out using conditions as follows. The MS worked with a capillary temperature equal to 275 °C and the source heater temperature set to 250 °C. The sheath gas flow was 50 units, while the auxiliary gas was set to 12 units. The source voltage was 3 kV. The capillary voltage and tube lens were −49 and −148 V, respectively. Elution was performed at a flow rate of 0.3 mL/min. The gradient started with 99% of 0.1% aqueous formic acid, isocratic conditions were maintained for 1 min, and then a 13-min linear gradient from 1% to 40% acetonitrile with 0.1% formic acid was applied. From 14 to 27 min the acidified acetonitrile was increased to 99%, followed by 2 min of 99% acetonitrile, and 6 min at the start conditions to re-equilibrate the column. Analyses were carried out using full scan mode, data-dependent MS^3^ scanning from *m*/*z* 100 to 1500, with collision induced dissociation (CID) equal to 35 (arbitrary units). Pure helium gas was used for CID.

In a second experimental framework, the MS worked with conditions optimized for rosmarinic acid analysis (experimental condition 2). Since the ionization of carnosic acid and carnosol was similar, rosmarinic acid (with diverse ionization/structure characteristics) was selected to optimize the secondary experimental condition in an attempt to cover a wider range of phenolic structures. The capillary temperature was set to 275 °C, while the source heater temperature was 50 °C. The sheath gas flow was 40 units, while auxiliary and sweep gas flow were set to 5 and 0 units, respectively. The source voltage was 4 kV. The capillary and tube lens voltage were −26 and −78 V, respectively. Analyses were carried out using full scan mode, data-dependent MS^3^ scanning from *m*/*z* 100 to 1500, with CID equal to 30 (arbitrary units). The chromatographic conditions were identical to those used for Experimental Conditions 1.

Quantification was performed in selected ion monitoring (SIM) mode by selecting the relative base peak at the corresponding mass to charge ratio (*m*/*z*) under Experimental Conditions 1.

### 3.3. HS-SPME/GC-MS Analysis

The volatile fraction composition of the rosemary extract sample was investigated according to the protocol of Cirlini et al. [33]. Briefly, 100 mg of rosemary extract were exactly weighted and placed in a 30 mL vial adding 20 µL of an aqueous toluene standard solution (348 mg/L). Sampling was performed in a thermostatted water bath at 40 °C for 35 min. During this time the sample was stirred at a constant speed and a fiber was inserted in the sample head space. For each SPME analysis, a silica fiber coated with 50/30 µm of divinylbenzene-carboxen-polydimethylsiloxane (DVB/Carboxen/PDMS, Supelco, Bellefonte, PA, USA) was used. After the sampling time, the fiber was removed from the vial and inserted into the GC-MS injector for the desorption of the volatiles over 2 min at 230 °C. The analysis was replicated twice.

All the analyses were performed on a Thermo Scientific Trace 1300 gas-chromatograph coupled to a Thermo Scientific ISQ MS equipped with an electronic impact (EI) source (Thermo Fisher Scientific Inc.). Separation was performed on a SUPELCOWAX 10 capillary column (Supelco, 30 m × 0.25 mm, f.t. 0.25 µm). All the injections were performed in splitless mode keeping the valve closed for 2 min. Temperature increase in the column was as follows: initiation at 50 °C for 3 min, increase to 200 °C at 5 °C per min, followed by a holding time of 12 min. The injector and transfer line temperatures were set at 230 °C and helium was used as carrier gas. Full scan mode was chosen as acquisition mode in the range of 41–500 *m*/*z*.

Peak identification was performed by comparing registered mass spectra with those present in the instrument library (NIST 14). LRIs were calculated for each detected signal on two different stationary phase columns, SUPELCOWAX 10 capillary column (Supelco, 30 m × 0.25 mm, f.t. 0.25 µm) and BP5MS (SGE Analytical Science, 30 m × 0.25 mm, f.t. 0.25 µm), according to retention times of a C_8_–C_20_ alkane standard solution analyzed under the same GC conditions applied for sample analyses. The semi-quantification of all detected GC signals was performed based on comparison to an internal standard (toluene).

## 4. Conclusions

This study described the phytochemical composition of a proprietary rosemary extract rich in carnosic acid with respect to its (poly)phenolic and volatile compounds. The use of an untargeted approach based on two chromatographic techniques coupled to mass spectrometry (UHPLC-ESI-MS^n^ and GC-MS) allowed elucidation of a broad array of compounds characterizing the phenolic and volatile fractions of this herb with multiple applications. This is, to our knowledge, the broadest profiling of rosemary secondary metabolites to date.

The UHPLC-ESI-MS^n−^-based characterization of the phenolic fraction of the rosemary extract allowed the tentative identification of 57 (poly)phenolic compounds belonging to different phenolic groups (24 flavonoids, 5 phenolic acids, 24 diterpenes, 1 triterpenic acid, and 3 lignans). Fourteen of these phenolic compounds are being described for the first time in this rosemary-based food ingredient. From a quantitative point of view, diterpenoids were the main class of (poly)phenolic structures, representing 97.2% of the phenolic content. With respect to the volatile fraction, 63 gas-chromatographic signals were detected and semi-quantified, describing the volatile profile and characteristics of this extract. The vast phytochemical characterization of this plant extract with food/pharma applications extends the number of molecules previously defined for rosemary and may assist in the study of their biological properties. This complete mass spectrometric analysis could be utilized to evaluate other rosemary-based products as well as other plant foodstuffs/extracts in order to fully unravel their phytochemical properties.

## Figures and Tables

**Table 1:** (Poly)phenolic compounds in rosemary extract identified by ultra-high performance liquid chromatography-mass spectrometry (UHPLC-MS^n^) in negative ionization mode under different mass spectrometry (MS) conditions.

ID.	Compounds	RT (min)	[M − H]^−^ (*m*/*z*)	MS^2^ ion Fragments (*m*/*z*) ^a^	MS^3^ ion Fragments (*m*/*z*) ^a^	Exp. 1 ^c^	Exp. 2 ^c^	Ident. ^d^
**1**	Caffeic acid	6.82	**179**	135		x	x	Std
**2**	Medioresinol	7.18	**387**	**207** ^b^, 163, 369	163	x	x	[4,12]
**3**	*p*-Coumaric acid	7.93	**163**	**119**	119	x	x	[4]
**4**	Luteolin-rutinoside	8.78	**593**	**285**	285, 241, 175, 199, 217	x	-	[4,22]
**5**	Luteolin-hexoside	8.98	**447**	**285**, 378	285, 241, 267, 199, 175	x	x	[4]
**6**	Isorhamnetin-3-*O*-hexoside	9.28	**477**	**315**, 300, 357, 462	300	x	x	[4]
**7**	4-hydroxybenzoic acid	9.47	**137**	93, 137		x	x	[4]
**8**	Apigenin-7-*O*-glucoside	9.82	**431**	**269**	225, 149, 201, 183, 281	x	x	[23]
**9**	Hesperidin (Hesperetin-7-*O*-rutinoside)	9.87	**609**	**301**	286, 242, 257, 283, 125	x	x	Std
**10**	Homoplantaginin (Hispidulin 7-glucoside)	10.04	**461**	**299**, 446, 284, 341	284, 255, 179	x	x	[3]
**11**	Rosmarinic acid	10.11	**359**	**161**, 179, 197, 223	161, 133	x	x	Std
**12**	Luteolin-7-*O*-glucuronide	10.28	**461**	**285**	241, 217, 175, 199	x	x	[4]
**13**	Dihydroxy-dimethoxyflavone derivative	10.33	**387**	**313**, 343	298	x	-	[4,24]
**14**	Dihydroxy-dimethoxyflavone	10.71	**313**	**298**	269, 283, 297, 280	x	-	[4,24]
**15**	Medioresinol derivative	11.22	**593**	**387**, 561, 519	207, 163, 369	x	x	[12]
**16**	Dihydroxy-dimethoxyflavone	11.24	**313**	**298**	283, 297, 269, 150	x	-	[4,24]
**17**	Luteolin-3′-acetyl-*O*-glucuronide	11.25	**503**	**285**, 399, 443	241, 243, 217, 199, 175	x	x	[13,23]
**18**	Medioresinol-glucuronide	11.37	**563**	**387**, 531, 489	207, 163, 369	x	x	[12]
**19**	Eriodictyol	11.46	**287**	**151**	107	x	x	[25]
**20**	Isorhamnetin-rutinoside	11.51	**623**	**315**, 300	300	x	x	[4,22]
**21**	Luteolin	11.75	**285**	285, 241, 199, 217, 257, 151, 179, 213		x	x	[4,22,26]
**22**	Isorhamnetin	11.91	**315**	**300**, 301, 287	300, 216, 228, 256, 272	x	x	[4,22]
**23**	Trihydroxy-methoxyflavone	11.98	**299**	**284**	283, 227, 256, 212, 200	x	x	[3,27]
**24**	Methyl rosmarinate	12.36	**373**	**179**, 135, 305	135	x	x	[26]
**25**	Apigenin-7-*O*-rutinoside	12.58	**577**	**269**, 307	269, 225, 201, 181, 149	x	x	[4,22]
**26**	Apigenin	13.02	**269**	**269**, 225, 149, 201, 183	181, 197, 169, 224	x	x	[4,22]
**27**	Hispidulin-rutinoside	13.21	**607**	**299**, 284, 269, 323	284	x	-	[3,27]
**28**	Hesperetin	13.41	**301**	**286**, 242, 257, 283, 125	258, 242, 199, 174, 215	x	x	[28]
**29**	5,6,7,10-tetrahydro-7-hydroxy rosmariquinone derivative	14.88	**345**	**301**	301, 258, 283, 273, 217	x	x	[14]
**30**	Cirsimaritin	14.98	**313**	**298**	283, 297, 269	x	x	[4,12]
**31**	Carnosol methyl ether isomer	15.35	**343**	**328**, 299	313, 299, 285	x	x	[14]
**32**	Rosmanol	15.46	**345**	**283**, 301, 327	268, 240, 227, 265, 239	x	x	Std
**33**	Rosmadial isomer or rosmanol quinone	15.97	**343**	**299**, 315	284, 243, 213, 256, 281	x	x	[3,4,13]
**34**	Rosmanol isomer (epirosmanol)	16.22	**345**	**283**, 301, 327	268, 227, 240, 239, 265	x	x	[3]
**35**	Carnosol quinone	16.27	**327**	**299**, 258	284, 271	x	x	[29]
**36**	Isosakuranetin	16.44	**285**	270, 229, 214, 201, 242		x	x	[25]
**37**	Genkwanin	16.45	**283**	**268**	268	x	x	[3,4]
**38**	Carnosic acid hexoside	16.76	**493**	**331**, 373, 313, 179	287, 244	x	x	Std
**39**	Rosmanol isomer (epiisorosmanol)	17.18	**345**	**301**	301, 286	x	x	[12]
**40**	5,6,7,10-tetrahydro-7-hydroxy rosmariquinone derivative	17.41	**345**	**301**	301, 258, 283, 273, 217	x	x	[14]
**41**	Carnosol methyl ether isomer	17.78	**343**	**299**, 328, 285, 343, 315	284, 243, 281, 299, 256	x	x	[14]
**42**	Carnosol methyl ether isomer	17.99	**343**	**328**, 313, 343, 299, 285	313, 300, 285, 257	x	x	[14]
**43**	Carnosic acid derivative	18.15	**455**	**331**, 287	287, 244	x	x	Std
**44**	Rosmanol methyl ether	18.59	**359**	**283**, 329, 300	268, 240, 227, 265, 239	x	-	[14]
**45**	Rosmadial or rosmanol quinone	18.62	**343**	**299**	243, 216, 284	x	x	[14]
**46**	Epiisorosmanol methyl ether	18.79	**359**	**315**	300	x	-	[14]
**47**	Rosmanol methyl ether isomer	18.96	**359**	**283**, 329, 300	268, 240, 227, 265, 239	x	x	[14]
**48**	Carnosol	19.07	**329**	**285**	270, 285, 269, 201, 214	x	x	Std
**49**	Carnosic acid quinone	19.51	**329**	**285**	270, 285, 201, 227	x	x	[30]
**50**	4′-Methoxytectochrysin	19.76	**297**	**282**, 269, 297, 254	267, 281, 238	x	x	[20]
**51**	Rosmadial	19.87	**343**	**315**, 299	287, 269, 297	x	x	[3,4,13]
**52**	Rosmaridiphenol	20.09	**315**	**285**, 179, 135	285, 214, 201, 270	x	x	[3,31]
**53**	5,6,7,10-tetrahydro-7-hydroxy rosmariquinone	20.37	**301**	**258**, 283, 273, 217, 233	243, 257, 188, 215, 162	x	x	[14]
**54**	Carnosic acid	20.85	**331**	**287**	287, 244, 272, 217	x	x	Std
**55**	12-*O*-Methylcarnosic acid	21.87	**345**	**301**, 286	286	x	x	Std
**56**	Carnosol isomer	21.88	**329**	329, 314, 299, 285		x	x	[31]
**57**	Betulinic acid	23.71	**455.5**	327, 317, 353, 409, 437		x	x	Std

^a^ Fragment ions are listed in order of relative abundance; ^b^ MS^2^ ions in bold were those subjected to MS^3^ fragmentation; ^c^ Exp. 1, detected under experimental condition 1 (carnosol), Exp. 2, experimental condition 2 (rosmarinic acid); ^d^ Ident., identification mode: [Reference] or Std (standard, compound identified by comparison of its retention time and MS data with that of a reference compound). Some compounds were defined as “derivatives” since parts of their spectra match those of their corresponding parent compounds but they cannot be fully elucidated.

**Table 2:** Quantitative results for rosemary extract (poly)phenolic compounds.

ID. ^a^	Compounds	Quantified as…	Concentration (mg/mL)		
**1**	Caffeic acid	Caffeic acid ^b^	0.03	±	0.00
**3**	*p*-Coumaric acid	Caffeic acid	0.01	±	0.00
**4**	Luteolin-rutinoside	Luteolin-4-glucoside	0.00	±	0.00
**5**	Luteolin-hexoside	Luteolin-4-glucoside	0.01	±	0.00
**6**	Isorhamnetin-3-*O*-hexoside	Rutin	0.04	±	0.00
**7**	4-hydroxybenzoic acid	Caffeic acid	0.01	±	0.00
**8**	Apigenin-7-*O*-glucoside	Vitexin (Apigenin-8-*C*-glucoside)	0.02	±	0.00
**9**	Hesperidin (Hesperetin-7-*O*-rutinoside)	Hesperidin (Hesperitin-7-rutinoside) ^b^	0.26	±	0.02
**10**	Homoplantaginin (Hispidulin 7-glucoside)	Luteolin-4-glucoside	0.12	±	0.02
**11**	Rosmarinic acid	Rosmarinic acid ^b^	0.12	±	0.01
**12**	Luteolin-7-*O*-glucuronide	Luteolin-4-glucoside	0.01	±	0.00
**13**	Dihydroxy-dimethoxyflavone derivative	Luteolin-4-glucoside	0.01	±	0.00
**14**	Dihydroxy-dimethoxyflavone	Luteolin-4-glucoside	0.00	±	0.00
**16**	Dihydroxy-dimethoxyflavone	Luteolin-4-glucoside	0.02	±	0.00
**17**	Luteolin 3′-*O*-acetyl-*O*-glucuronide	Luteolin-4-glucoside	0.01	±	0.00
**20**	Isorhamnetin rutinoside	Rutin	0.00	±	0.00
**21**	Luteolin	Luteolin-4-glucoside	0.14	±	0.03
**22**	Isorhamnetin	Rutin	0.12	±	0.01
**23**	Trihydroxy-methoxyflavone	Vitexin (Apigenin-8-*C*-glucoside)	0.18	±	0.01
**24**	Methyl rosmarinate	Rosmarinic acid	0.02	±	0.00
**25**	Apigenin-7-*O*-rutinoside	Vitexin (Apigenin-8-*C*-glucoside)	0.00	±	0.00
**26**	Apigenin	Vitexin (Apigenin-8-*C*-glucoside)	0.55	±	0.04
**27**	Hispidulin-rutinoside	Luteolin-4-glucoside	0.89	±	0.15
**29**	5,6,7,10-tetrahydro-7-hydroxyrosmariquinone derivative	Carnosol	0.27	±	0.02
**31**	Carnosol methyl ether isomer	Carnosol	0.00	±	0.00
**32**	Rosmanol	Rosmanol ^b^	0.15	±	0.01
**33**	Rosmadial isomer or rosmanolquinone	Rosmanol	0.00	±	0.00
**34**	Rosmanol isomer (epirosmanol)	Rosmanol	0.14	±	0.01
**35**	Carnosol quinone	Carnosol	0.02	±	0.00
**38**	Carnosic acid hexoside	Carnosic acid	0.00	±	0.00
**39**	Rosmanol isomer (epiisorosmanol)	Rosmanol	0.06	±	0.01
**40**	5,6,7,10-tetrahydro-7-hydroxyrosmariquinone derivative	Carnosol	0.08	±	0.01
**41**	Carnosol methyl ether isomer	Carnosol	0.00	±	0.00
**42**	Carnosol methyl ether isomer	Carnosol	0.00	±	0.00
**43**	Carnosic acid derivative	Carnosic acid	0.00	±	0.00
**44**	Rosmanol methyl ether	Rosmanol	0.00	±	0.00
**45**	Rosmadial or rosmanol quinone	Rosmanol	0.89	±	0.08
**46**	Epiisorosmanol methyl ether	Rosmanol	0.01	±	0.00
**47**	Rosmanol methyl ether isomer	Rosmanol	0.00	±	0.00
**48**	Carnosol	Carnosol ^b^	28.89	±	2.24
**49**	Carnosic acid quinone	Carnosic acid	0.17	±	0.14
**51**	Rosmadial	Rosmanol	1.25	±	0.07
**52**	Rosmaridiphenol	Carnosol	0.57	±	0.04
**53**	5,6,7,10-tetrahydro-7-hydroxyrosmariquinone	Carnosol	0.01	±	0.00
**54**	Carnosic acid	Carnosic acid ^b^	121.08	±	7.67
**55**	12-*O*-Methylcarnosic acid	12-*O*-Methylcarnosic acid	6.90	±	0.58
**56**	Carnosol isomer	Carnosol ^b^	1.16	±	0.07
**57**	Betulinic acid	Betulinic acid ^b^	2.10	±	0.25
		*Hydroxybenzoic acids* ^c^	0.01	±	0.00
		*Hydroxycinnamic acids* ^d^	0.04	±	0.00
		*Rosmarinic acid derivatives* ^e^	0.14	±	0.01
		*Flavones* ^f^	1.82	±	0.18
		*Flavonols* ^g^	0.31	±	0.02
		*Flavanones* ^h^	0.26	±	0.02
		*Carnosic acid derivatives* ^i^	128.15	±	8.11
		*Carnosol derivatives* ^j^	30.08	±	2.31
		*Rosmanol derivatives* ^k^	1.25	±	0.11
		*Other diterpene derivatives* ^l^	2.18	±	0.12
		*Triterpenic acids* ^m^	2.10	±	0.25
		*Total phenolics*	166.32	±	11.05

^a^ See Table 1 for peak assignment; ^b^ Quantified by comparison with its corresponding standard; ^c^ Hydroxybenzoic acids include compound **7**; ^d^ Hydroxycinnamic acids, compounds **1** and **3**; ^e^ Rosmarinic acid derivatives, compounds **11** and **24**; ^f^ Flavones, compounds **4**, **5**, **8**, **10**, **12**–**14**, **16**, **17**, **23**, and **25**–**27**; ^g^ Flavonols, compounds **6** and **20**–**22**; ^h^ Flavanones, compound **9**; ^i^ Carnosic acid derivatives, compounds **38**, **43**, **49**, **54**, and **55**; ^j^ Carnosol derivatives, compounds **31**, **35**, **41**, **42**, **48**, and **56**; ^k^ Rosmanol derivatives, compounds **32**–**34**, **39**, and **44**–**47**; ^l^ Other diterpene derivatives, compounds **29**, **40**, and **51**–**53**; and ^m^ Triterpenic acids, compound **57**. Mean (*n* = 3) ± standard deviation(SD).

**Table 3:** Identification of rosemary extract volatile compounds, with relative aromatic notes, calculated linear retention indices (LRIs) on two different stationary phases (“wax” polar and “BP5” a-polar), identification methods, references, and relative amounts (mean ± SD).

ID.	Identification	Flavor Note (Flavornet.org)	LRI-wax	LRI-BP5	Identif. Method	Reference	Concentration (µg/g)
**1**	1R-α-Pinene	Intense woody, pine	**1022**	**928**	MS + LRI	[34]	4.34 ± 0.65
**2**	Hexanal	Green	**1087**	**776**	MS + LRI	[35]	2.81 ± 0.28
**3**	α-Thujene	Woody	**1128**	**948**	MS + LRI	[34]	76.26 ± 13.13
**4**	β-Myrcene	Peppery, terpenic	**1170**	**983**	MS + LRI	[34]	6.36 ± 0.91
**5**	(+)-4-Carene		**1185**	**1080**	MS		15.96 ± 2.11
**6**	Heptanal	Fresh, aldehydic	**1194**	**890**	MS + LRI	[36]	4.90 ± 0.44
**7**	D-Limonene	Sweet, citrus, peely	**1205**	**1024**	MS + LRI	[35]	11.78 ± 2.80
**8**	Eucalyptol	Eucalyptus, herbal	**1213**	**1025**	MS + LRI	[34]	20.22 ± 2.58
**9**	Cosmene	Dahlia, *Laurus nobilis*	**1223**	**998**	MS + LRI	[33]	3.39 ± 0.28
**10**	Not Identified		**1231**	**984**			5.88 ± 1.36
**11**	2-Pentylfuran	Fruity	**1239**		MS + LRI	[37]	3.01 ± 0.79
**12**	γ-Terpinene	Terpy, citrus	**1251**	**1052**	MS + LRI	[35]	6.26 ± 1.17
**13**	3-Octanone	Mushroom, ketonic, cheesy and moldy	**1261**		MS + LRI	[37]	0.61 ± 0.19
**14**	o-Cymene	Lavender and cypress oil	**1276**	**1017**	MS + LRI	[33]	15.14 ± 1.87
**15**	α-Terpinene	Terpy, woody,	**1287**	**1011**	MS + LRI	[36]	5.93 ± 0.67
**16**	1-Octen-3-one	Intense creamy, earthy	**1308**		MS + LRI	[34]	0.44 ± 0.28
**17**	2,4-Hexadienal	Green, creamy	**1323**		MS + LRI	[38]	0.48 ± 0.09
**18**	2-Heptenal	Green, fatty	**1331**		MS + LRI	[37]	2.58 ± 0.44
**19**	6-Methyl-5-hepten-2-one	Citrus	**1344**		MS+LRI	[37]	1.06 ± 0.32
**20**	3-Octanol	Musty, mushroom	**1396**		MS		0.74 ± 0.14
**21**	Nonanal	Waxy, aldehydic	**1400**	**1094**	MS + LRI	[35]	3.47 ± 0.91
**22**	(E)-2-Octenal	Fatty, green, herbal	**1437**	**1048**	MS + LRI	[37]	2.83 ± 0.59
**23**	Ethyl caprylate	Fruity, waxy	**1441**		MS + LRI	[39]	7.66 ± 2.43
**24**	p-Cymenene	Phenolic	**1445**		MS		34.70 ± 5.71
**25**	Ylangene		**1487**	**1369**	MS + LRI	[40]	8.06 ± 1.50
**26**	α-Copaene	Woody, spicy, honey	**1495**	**1374**	MS + LRI	[37]	1.02 ± 0.30
**27**	trans-2,4-Heptadienal	Sweet creamy, fatty	**1503**		MS + LRI	[37]	0.77 ± 0.10
**28**	Camphor	Camphoreous	**1524**		MS + LRI	[39]	41.52 ± 6.00
**29**	2-Nonenal	Fatty, green, melon	**1543**		MS + LRI	[35]	0.31 ± 0.14
**30**	β-Linalool	Floral	**1553**	**1092**	MS + LRI	[33]	18.79 ± 3.38
**31**	Isopulegol	Minty, herbaceous	**1570**		MS		0.37 ± 0.09
**32**	Pinocarvone	Minty	**1576**	**1154**	MS + LRI	[41]	3.56 ± 0.56
**33**	Bornyl acetate	Camphoreous, woody	**1590**	**1278**	MS + LRI	[42]	54.02 ± 8.77
**34**	β-Caryophyllene	Spicy, peppery	**1604**	**1420**	MS + LRI	[37]	26.44 ± 4.84
**35**	Terpinen-4-ol	Peppery, woody	**1608**	**1174**	MS + LRI	[34]	16.48 ± 3.65
**36**	Hotrienol	Sweet, tropical	**1616**	**1105**	MS + LRI	[33]	1.42 ± 0.76
**37**	α-Thujenal		**1638**		MS		1.39 ± 0.27
**38**	Ethyl caprate	Sweet, waxy	**1646**	**1385**	MS + LRI	[39]	12.41 ± 1.93
**39**	Humulene	Woody	**1654**	**1456**	MS + LRI	[43]	2.16 ± 0.38
**40**	α-Caryophyllene	Woody, spicy, earthy	**1677**	**1404**	MS+LRI	[44]	38.53 ± 7.24
**41**	α-Muurolene		**1697**	**1478**	MS + LRI	[45]	9.57 ± 1.98
**42**	α-Terpineol	Pine, lilac, citrus	**1704**		MS + LRI	[46]	24.70 ± 4.46
**43**	Borneol	Pine, woody, camphoreous	**1708**	**1165**	MS + LRI	[47]	11.92 ± 2.01
**44**	Verbenone	Camphor, menthol	**1720**	**1203**	MS + LRI	[34]	77.59 ± 12.85
**45**	τ-Elemene		**1730**		MS		4.00 ± 0.96
**46**	p-Methen-3-one		**1737**	**1246**	MS		2.57 ± 0.58
**47**	Carvone	Minty, licorice	**1743**	**1213**	MS		0.89 ± 0.23
**48**	δ-Cadinene	Thyme, herbal, woody	**1763**	**1517**	MS + LRI	[34]	4.20 ± 1.04
**49**	Myrtenol	Minty, camphoreous	**1798**	**1315**	MS + LRI	[41]	0.76 ± 0.15
**50**	2-Phenylethyl acetate	Floral	**1826**		MS + LRI	[39]	0.98 ± 0.12
**51**	Calamenene	Herb spice	**1840**		MS + LRI	[48]	1.76 ± 0.46
**52**	p-Cymen-8-ol	Sweet, fruity, coumarinic	**1857**	**1183**	MS + LRI	[33]	3.06 ± 0.80
**53**	2-Phenyl ethanol	Floral, rose	**1920**		MS + LRI	[39]	1.00 ± 0.22
**54**	α-Calacorene	Woody	**1925**		MS + LRI	[49]	2.46 ± 0.63
**55**	Eucarvone	Minty	**1933**		MS		8.52 ± 2.22
**56**	5,5-Dimethyl-1-ethyl-1,3-cyclopentadiene		**1971**	**984**	MS		0.78 ± 0.25
**57**	5,5-Dimethyl-1-ethyl-1,3-cyclopentadiene-like		**2008**		MS		1.93 ± 0.49
**58**	Eugenol methyl ether	Sweet, spicy, cinnamon	**2022**		MS		1.29 ± 0.43
**59**	2-Ethylcyclohexanone		**2095**		MS		0.58 ± 0.13
**60**	Eugenol	Spicy	**2165**	**1345**	MS + LRI	[33]	4.19 ± 1.11
**61**	Thymol	Herbal	**2180**	**1293**	MS + LRI	[33]	0.36 ± 0.13
**62**	p-Thymol		**2195**		MS		0.46 ± 0.11
**63**	Carvacrol	Spicy	**2205**		MS + LRI	[33]	0.73 ± 0.19

## Abbreviations

The following abbreviations are used in this manuscript:

CID	collision-induced dissociation
EI	electronic impact
ESI	electrospray ionisation
GC-MS	gas chromatography-mass spectrometry
HS-SPME	head space solid-phase microextraction
LRI	linear retention indices
MS	mass spectrometry
SIM	selected ion monitoring
UHPLC-ESI-MS^n^	ultra-high performance liquid chromatography-electrospray ionization-mass spectrometry
